# The impact of pertussis in infants: insights from a hospital-based enhanced surveillance system, Lazio region, Italy, 2016 to 2019

**DOI:** 10.2807/1560-7917.ES.2021.26.24.2000562

**Published:** 2021-06-17

**Authors:** Elisabetta Pandolfi, Francesco Gesualdo, Caterina Rizzo, Luisa Russo, Ilaria Campagna, Emanuela Carloni, Carlo Concato, Giulia Linardos, Alberto Villani, Sara Ciampini, Antonino Reale, Elena Boccuzzi, Fabio Midulla, Alberto E Tozzi

**Affiliations:** 1 Bambino Gesù Children’s Hospital, IRCCS, Multifactorial and Complex Disease Research Area, Rome, Italy; 2 Bambino Gesù Children’s Hospital, IRCCS, Virology Unit, Rome, Italy; 3 Bambino Gesù Children’s Hospital, IRCCS, Paediatric and Infectious Diseases Unit, Rome, Italy; 4 Bambino Gesù Children’s Hospital, IRCCS, Emergency Department, Rome, Italy; 5 Sapienza University, Department of Paediatrics, Rome, Italy

**Keywords:** pertussis, epidemiology, Public Health Surveillance, Vaccine-preventable diseases

## Abstract

**Background:**

Routine surveillance systems for pertussis often suffer from under-recognition and under-reporting.

**Aim:**

Our aim was to describe the epidemiology and the clinical features of pertussis in children younger than 1 year in an Italian region, detected through an enhanced hospital surveillance system.

**Methods:**

From 2016 to 2019, we monitored the incidence and the clinical characteristics of hospitalised pertussis cases younger than 1 year in two paediatric hospitals involved in the PERTINENT project.

**Results:**

We detected 141 pertussis cases, corresponding to an estimated incidence of 105.8 per 100.000 in 2016, 91.7 per 100.000 in 2017, 64.5 per 100.000 in 2018 and 40.9 per 100.000 in 2019, based on the hospitals’ catchment area, roughly corresponding to the Lazio region. A total of 101 cases (77.1%) had a household member with cough or other respiratory symptoms. The most frequent combination of symptoms was paroxysmal cough with apnoea in the absence of fever. Almost 40% had been prescribed an antibiotic treatment before hospitalisation, and the median time from symptom onset to contact with the hospital was 8 days. Thirty-one (22.0%) had complications.

**Conclusion:**

An enhanced surveillance system showed a high incidence of pertussis among infants in the Lazio region, where the impact of this disease may still be underestimated. Increasing the coverage of pertussis immunisation among pregnant women and improving the capacity for early detection in primary care may contribute to reducing the impact of pertussis among infants.

## Introduction

Pertussis in infants is frequently associated with severe clinical pictures [1]. Unvaccinated infants pay the highest toll in terms of morbidity and mortality for pertussis [2]. The first dose of the pertussis vaccine is usually administered at 2 or 3 months of age and infants are not protected until completion of the first three doses by 11 months of age [3,4].

A deep understanding of the epidemiology of the disease through surveillance is needed to inform effective control measures.

Pertussis incidence figures are strongly heterogeneous across different countries [5-8] and are often not consistent with seroepidemiological studies, which have hypothesised that the true incidence of the disease might be much higher than reported by surveillance systems [9,10].

The difference in incidence may not reflect a true difference in the disease occurrence, as incidence figures can be influenced by a number of factors, mainly related to under-recognition and under-reporting [6,11]. Firstly, although the introduction of the RT-PCR laboratory test for confirmation of the diagnosis has recently improved the sensitivity of pertussis surveillance systems in Europe [5], the suspicion of pertussis and the subsequent decision to prescribe a laboratory confirmation test are often based on non-specific clinical signs, which may lead clinicians to suspect other respiratory conditions. Secondly, the pertussis case definitions from the European Centre for Disease Prevention and Control (ECDC) includes prolonged cough [12], which also guides the diagnosis of suspected pertussis. However, based on our clinical experience and on the results shown below, parents of infants often seek care well before 2 weeks from the start of the cough and therefore, cases can easily be misdiagnosed, with a consequent impact on disease reporting.

In order to better inform surveillance and case management strategies, it may be useful to study the epidemiology of the disease and combine incidence data with the factors associated with access to healthcare and with information on the clinical features and the course of the disease.

In 2015, the ECDC funded the *Pertussis in Infants European Network* (PERTINENT), a hospital-based active sentinel surveillance system to measure the incidence of whooping cough in infants under the age of than 1 year in Europe. PERTINENT currently includes 37 hospitals from seven surveillance sites in the European Union and European Economic Area, including two large paediatric hospitals located in the Lazio region, Italy.

In Italy, the primary immunisation schedule for pertussis includes three doses in the 3rd, 5th and 11th month of age, with booster doses recommended in the 6th year of life, between the 12th and 18th year of life, and every 10 years after that [13]. Since 2017, the Ministry of Health has recommended pertussis immunisation in pregnancy, which has not achieved a high coverage yet.

In this study, we take advantage of the PERTINENT surveillance system to describe the epidemiology of pertussis in an Italian region, its clinical features and the factors affecting the timing of access to healthcare services among patients with pertussis in the first year of life.

## Methods

### Study design and setting

This is an observational study in a population of infants younger than 1 year, hospitalised for pertussis and identified through an enhanced hospital surveillance programme. The study was conducted between 1 January 2016 and 31 December 2019 and included two large hospitals in the metropolitan area of Rome, Lazio region, Italy.

### Study population

Based on the PERTINENT protocol [14], the study population included all infants younger than 1 year accessing the emergency room with the following case definition: apnoea or a cough associated with at least one additional of the following signs: paroxysms, whoop or post-tussive vomiting (‘typical’ presentation). Patients with none of these typical symptoms were included in the study population if their physician had suspected pertussis (‘atypical’ presentation).

Patients with these criteria were screened for pertussis with RT-PCR and, when possible, bacterial culture of the nasopharyngeal aspirate. The patients’ families were interviewed after signing an informed consent form. We defined a confirmed pertussis case as a patient meeting the above case definition and either a positive RT-PCR for *Bordetella pertussis* or a positive culture.

### Data collection

We collected the following data through an interview with cases’ parents: sociodemographic data, gestational age, birth weight, level of education and employment of the parents, patient’s immunisation status against pertussis through vaccination cards, date of symptom onset, kind of feeding at symptom onset, number of household members and presence of a household member with respiratory symptoms. We also collected the following information during admission: length of stay, complications and admission to the intensive care unit. We also recorded if the patient had both leukocytosis (i.e. white blood cell counts greater than the maximum value for age [15]) and a > 50% proportion of lymphocytes over the total leukocyte count.

### Laboratory confirmation

Nasopharyngeal aspirates were performed and processed using a standardised protocol [14]. Samples were collected within 24 h of hospital admission and processed immediately or stored at −70 °C until performing the test.

Nasopharyngeal aspirates were immediately spread onto Bordet–Gengou and Regan–Lowe selective agar after being homogenised through vortexing. The plates were incubated in a humidified incubator at 37 °C for 7 days. Colonies of *B. pertussis* and *B. parapertussis* were verified by Gram staining, biochemical tests and MALDI-TOF mass spectrometry.

Nucleic acids were extracted from a 200 μL sample of rhinopharyngeal aspirate and purified using the EZ1 Virus Mini Kit v. 2.0 on the EZ1 Advanced XL platform (Qiagen, GmbH, Hilden, Germany). Nucleic acid extracts are eluted into 90 μL of buffer and processed immediately. The presence of *B. pertussis* was investigated using a Bordetella Real Time PCR kit targeting IS*481* (Bordetella R-gene assay Argene, Biomerieux, Marcy l’Etoile, France). To prevent misdiagnosis of *B. holmesii* as *B. pertussis*, all samples positive for *B. pertussis* are confirmed with a specific real-time PCR assay for *B. pertussis* using the promoter of pertussis toxin (*ptxP*) gene. We also used the Bordetella Real Time PCR kit (Bordetella Parapertussis R-gene assay Argene, Biomerieux) targeting IS*1001* for *B. holmesii* and B. *bronchiseptica*.

### Statistical analysis

We estimated pertussis incidence related to the resident population younger than 1 year in the catchment area of the two hospitals [16]. This catchment area was arbitrarily defined as the provinces generating pertussis cases for the period under surveillance.

We compared sociodemographic and clinical characteristics of pertussis cases with patients with a negative RT-PCR. Data are presented as mean and standard deviation (SD), or median and range, or proportion and 95% confidence intervals (CI), as appropriate. Differences in proportions were evaluated through Fisher's exact test or chi-squared test, as appropriate. The time lag from symptom onset to access to the emergency room and associated factors were studied through Cox proportional hazard models.

Stata 13 software (StataCorp, Texas, United States) was used for statistical analysis.

### Ethical statement

The study was approved by the Bambino Gesù Children’s Hospital Ethical Committee (protocol no. 1064_OPBG_2016) and was conducted according to the Declaration of Helsinki, 2013 [17].

## Results

### Characteristics of infant pertussis cases and their families

A total of 153 infants under 1 year of age were hospitalised with laboratory-confirmed pertussis in the period between 1 January 2016 and 31 December 2019. Twelve were excluded for the following reasons: one patient was excluded because of a time lag of more than 90 days between onset of symptoms and nasopharyngeal aspirate and 11 did not meet the PERTINENT protocol’s case definition. Of these, one had no respiratory symptoms but was swabbed for pharyngeal hyperaemia, one had only a cough, four had only a fever, four had both a cough and a fever, and one had a cough and cyanosis; in none of the cases had the family paediatrician suspected pertussis. A total of 141 infants with a positive RT-PCR for *B. pertussis* were finally included in the analysis. Ninety-three of them (65.9%) also had a positive pertussis culture. No other *Bordetella* species were detected.

Table 1 presents sociodemographic characteristics of all patients screened for pertussis, by RT-PCR results. There was a slightly higher proportion of male cases (55.3%). The sociodemographic level was generally high, 12% were preterm infants and nearly 70% had siblings.

**Table 1:** General characteristics of infant pertussis cases, Lazio, Italy, January 2016−December 2019 (n = 141)

	Positive PCR (n = 141)			Negative PCR (n = 405)			p value
	Median		Range	Median		Range	
Age in months	2.6		0.4–11.6	2.1		0.3–12.0	0.730
Birth Weight in kg	3.2		0.700–4.925	3.1		0.690–5.020	0.002
Gestational age in weeks	39		27–41	38		26–42	0.005
	**n**	**%**	**95% CI**	**n**	**%**	**95% CI**	**p value**
Female	63	44.7	36.6–52.9	196	48.4	43.4–53.4	0.447
Preterm infants	17	12.1	7.2–18.6	84	20.7	16.9–25.0	0.022
Working mother	82	58.6	49.9–66.0	236	58.3	53.3–63.1	0.951
Mother with degree	47	33.8	25.9–41.4	145	36.3	31.5–41.2	0.605
Working father	131	93.6	87.7–96.3	380	94.1	88.1–97.0	0.835
Father with degree	39	28.3	20.7–35.5	105	26.3	22.0–30.9	0.645
More than three household members	105	74.5	66.8–81.1	317	78.3	73.9–82.2	0.353
One or more siblings	96	68.1	60.0–75.4	305	75.3	70.8–79.4	0.094
Mother received pertussis vaccine during pregnancy	0	0	NA	3	0.7	0.2–2.2	0.814

CI: confidence interval; NA: not applicable; n.c. not calculable.

Denominators vary because of missing data for the following variables: working mother, mother with degree, working father, father with degree.

Fifty-five of 141 cases (39.0%) were aged 2 months or younger and were not, therefore, eligible for immunisation. Among the 86 infants eligible for immunisation, 42 had not received any dose of the vaccine, 36 had received only one dose, eight had received two doses and none had received three doses. None of the mothers of cases had received pertussis immunisation during pregnancy. Among the 131 cases for whom this information was available, 101 (77.1%) had one or more relatives with cough or other respiratory symptoms. The most frequent categories were siblings (n = 52; 39.7%) and mothers (n = 32; 24.4%). Only two of 52 infant cases with symptomatic siblings had siblings who had not received any dose of pertussis immunisation, while symptomatic siblings of 44 of 52 cases had received at least three doses.

Compared with non-cases (i.e. patients with a non-pertussis respiratory infection), pertussis cases were born at a significantly higher gestational age and with a higher birth weight and were less frequently preterm.

### Epidemiology

The distribution of cases by month and year is reported in the Figure. We observed a peak in incidence rates in 2016 with a subsequent decreasing trend.

**Figure:**
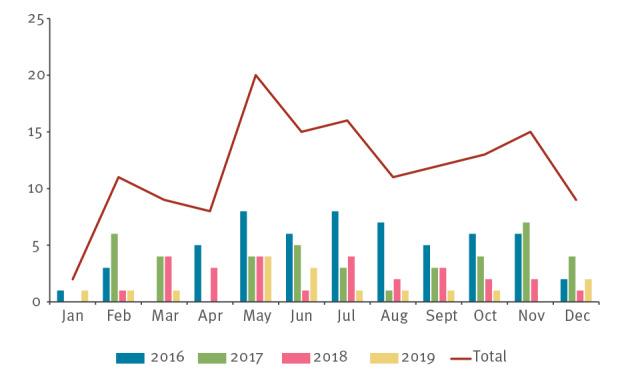
Cases of pertussis in infants by year and month, Lazio, Italy, January 2016−December 2019 (n = 141)

The catchment area of the two hospitals, defined as the provinces generating cases in the study period, included all provinces of the Lazio region, except for the province of Rieti, which accounted for less than 2.5% of the population younger than 1 year in the whole region. Therefore, in the rest of the paper, to perform comparisons with official surveillance data (which are reported by region and not by province) we will consider the catchment area of the two hospitals as an area roughly corresponding to the whole Lazio region. Based on the catchment area, the estimated incidence was 105.8 per 100.000 in 2016, 91.7 per 100.000 in 2017, 64.5 per 100.000 in 2018 and 40.9 per 100.000 in 2019. The majority of cases were observed between May and September, with lower figures in winter.

### Clinical presentation

Cough, paroxysmal cough and apnoea were the most frequent symptoms (Table 2). The most frequent pattern of symptoms was a combination of paroxysmal cough and apnoea in the absence of fever. These three criteria were simultaneously recorded in 76 (53.9%) cases. Forty-three (31%) cases had leukocytosis and lymphocytosis. A total of 56 infants (39.7%) had received an antibiotic prescription before admission to the hospital. Of these, 30 were prescribed a macrolide, while the remaining 26 received either amoxicillin or an oral cephalosporin.

**Table 2:** Symptoms observed in infant pertussis cases, Lazio, Italy, January 2016−December 2019 (n = 141)

	Positive PCR (n = 141)			Negative PCR (n = 405)			p
	n	%	95% CI	n	%	95% CI	
Cough	136	96.5	92.3–98.7	332	82.0	77.9–85.6	< 0.001
Paroxysmal cough	120	85.1	78.5–90.2	203	50.1	45.1–55.1	< 0.001
Apnoea	107	75.9	68.3–82.4	194	47.9	42.9–52.9	< 0.001
Cyanosis	80	56.7	48.5–64.7	105	26.1	21.8–30.6	< 0.001
Whooping	77	54.6	46.3–62.7	58	14.9	11.5–18.8	< 0.001
Post-tussive vomiting	64	45.4	37.3–56.7	151	37.3	32.6–42.2	0.090
WBC > max for age and LYM > 50%	43	31.2	23.8–39.2	28	7.2	4.8–10.2	< 0.001
Fever	32	22.7	16.3–30.1	178	44.0	39.1–48.9	< 0.001
Conjunctival haemorrhage	15	10.6	6.3–16.7	15	3.7	2.1–6.1	0.002
Petechiae	14	10.0	5.8–15.8	16	4.0	2.3–6.5	0.008
Mother with symptoms	32	24.4	17.3–32.7	75	19.8	15.9–24.2	0.267

CI: confidence interval; WBC: white blood cells. LYM: lymphocytes.

Denominators vary because of missing data for the following variables: cyanosis, whooping, WBC > max for age and LYM > 50%, conjunctival haemorrhage, petechiae, mother with symptoms.

Cough, paroxysmal cough, apnoea, cyanosis, whooping, leukocytosis and lymphocytosis, conjunctival haemorrhage and petechiae were significantly more frequent in pertussis cases compared with non-cases. Fever was more frequently reported among non-cases.

### Clinical course during admission

The median time to access the emergency room after symptom onset was 8 days (range: 1–53; interquartile range: 3–14). Apnoea and having had an antibiotic prescription for the current respiratory infection were significantly associated with a shorter time between symptom onset and hospitalisation in the emergency room (apnoea: hazard ratio (HR) = 0.61, 95% CI: 0.40–0.93; antibiotics: HR = 0.55, 95% CI: 0.38–0.80). The median length of stay was 7 days (range: 1–111).

Thirty-one children (22.0%) had complications during admission, including the following: hypoxaemia (n = 13), difficult feeding (n = 11), dehydration (n = 3), bacterial superinfection (n = 2), seizures (n = 2), cerebral haemorrhage (n = 1), rectal prolapse (n = 1), rib fractures (n = 1) and acute renal failure (n = 1). Nine cases were admitted to the intensive care unit, none died.

The clinical presentation of the disease was similar in term and preterm infants, although complications were more frequent in the latter group. Specifically, dehydration (2/17 vs 1/124, p < 0.01) and convulsions (2/17 vs 0/124, p < 0.01) were observed more frequently in preterm infants with pertussis.

## Discussion

Studying the epidemiology and the impact of pertussis in infants younger than 1 year is crucial to plan appropriate prevention strategies at the local level. Through enhanced surveillance, we found an incidence of pertussis in an Italian region that was higher than that estimated from a sentinel surveillance system in Italy in 2008 [18]. Although pertussis immunisation coverage in Italy exceeds 95% in the first 2 years of life [19] and the Ministry of Health has recommended immunisation in pregnancy since 2017 [13], pertussis cases still occur among infants too young to be vaccinated.

Although pertussis vaccination coverage across Europe is relatively homogeneous, incidence figures in Europe vary, mainly owing to differences in surveillance systems, case definitions and methods for diagnosis, which consequently affect disease reporting. In one study examining surveillance data from several European countries in 2017, the largest number of pertussis cases were reported in Germany, the Netherlands, Poland, Spain and the United Kingdom (UK), but there were substantial variations across countries [5]. In the same report, infants were the most frequently affected age group, with the exception of the Netherlands, Slovenia and Norway. According to ECDC data, European countries had in 2017 an average incidence of 53.9 cases per 100,000 in infants below 1 year of age, with 50% of cases among infants aged 3 months or younger [8]. The incidence estimated in infants in our study was 91.7 per 100,000 in 2017, i.e. nearly twice as high, in the same year. Routine surveillance data for the Lazio region are available for the 0–3 year-old group only and indicate an incidence of 31.7 and 29.0 per 100.000 in 2016 and 2017, respectively [20].

If the results of our study are applied to the rest of the country, it is likely that Italy is impacted by significant under-reporting for pertussis. The temporal trend of pertussis showed an incidence peak in 2016 with a decreasing trend in the following years. The peak (and its subsequent decline) was not associated with outbreaks or with major modification of immunisation policies. Therefore, it can be interpreted as a normal variation in the usual frame of the pertussis epidemic cycle [18].

Twelve per cent of the enrolled population were preterm babies. Taking into account that 7% of Italian children are estimated to be born preterm [21], there is an over-representation of this subgroup among infants hospitalised for pertussis. Although this result should be interpreted with caution given the wide 95% CI of the proportion, it is in line with previous observations conducted on larger populations in the UK (10.6% prevalence of preterm infants among infants hospitalised for pertussis) [22] and in Norway (10%) [23]. The clinical picture of preterm infants was slightly worse compared with term infants, with a higher incidence of dehydration and seizures, while other symptoms, other complications and the length of stay were similar between the two groups.

Under-recognition of pertussis is also an issue. Although a significant proportion of cases in our study presented with symptoms commonly associated with pertussis, others had non-specific signs that resembled other respiratory infections. Moreover, almost 40% of enrolled cases accessed the emergency room after a symptom duration shorter than 1 week. Interestingly, more than one third of pertussis cases had received an antibiotic prescription before accessing the emergency room, which was a macrolide in only ca 50% of cases. This may indicate that in almost half of the infants on antibiotic treatment, the physician had not suspected a diagnosis of pertussis. These data are in line with the small likelihood of suspecting pertussis detected in a sample of Italian paediatricians and physicians back in 2013 [24]. Moreover, the severity of the clinical picture affected the time from symptom onset to access to the emergency room, as this time was shorter in infants with apnoea or an antibiotic prescription.

Early recognition of pertussis may benefit from the availability of tools for pertussis diagnosis in primary care. Point-of-care diagnostic tests are not commonly available yet, but ongoing research in this field is promising [25]. The potential clinical impact of tools for point-of-care diagnosis in primary care is well recognised [26], as they may improve the estimation of the impact of pertussis and the management of the disease, avoiding unnecessary antibiotic prescription in case of viral infections and allowing early, appropriate antibiotic treatment to prevent secondary cases.

The impact of pertussis observed in this study may be at least partially preventable. As shown in other studies, the efficacy estimate for only one dose of pertussis vaccine is limited. Moreover, timeliness of immunisation start is crucial, as, according to our results, a large proportion of cases had not yet started their vaccination course although they were eligible. Immunisation during pregnancy has been proven to be safe, effective and cost-effective in preventing pertussis hospitalisations in infants [27,28]. Although the Italian vaccine programme includes a recommendation for immunising pregnant women against pertussis in the third trimester, ideally in the 28th gestational week [13], data on immunisation coverage are not available yet. In 2016 and 2017, a small-scale seroepidemiological study showed non-protective titres of antibodies in all tested pregnant women in the south of Italy [29]. Two surveys conducted between 2015 and 2018 showed that fewer than 2% of interviewed women had received tetanus, diphtheria and acellular pertussis (Tdap) vaccination during pregnancy, and only one third showed willingness to be vaccinated during pregnancy [30,31]. Based on these studies, we assume that Tdap vaccination coverage during pregnancy is still poor in Italy. Taking into account the costs of hospitalisation and the fact that severe pertussis cases mostly occur in children too young to be eligible for vaccination, immunisation in pregnancy remains a mainstay of pertussis prevention and should be urgently implemented and promoted, given its efficacy, safety and cost-effectiveness and that it had a higher impact than a cocooning strategy or immunisation before pregnancy [27,32]. Nevertheless, a pertussis vaccination programme in pregnancy may have a limited impact on preventing pertussis among preterm infant (over-represented among infants hospitalised with pertussis), because the opportunity for maternal vaccination is smaller in this particular group, as previously demonstrated in a study conducted in the UK. For this reason, from 2016, the recommendation for maternal vaccination in the UK has been anticipated to be between 20 and 32 weeks of gestational age [22]. In Italy, the timing of the pertussis vaccination in pregnancy may be reviewed accordingly.

This study has a number of limitations. Based on the home addresses of the enrolled patients, we assumed that the catchment area of the participating hospitals roughly corresponded to the whole Lazio region. However, we cannot exclude that our study missed cases who might have accessed other hospitals. In both cases, the real incidence in the region could be higher than what we recorded. Unfortunately, recent regional data by age group are not available, therefore reliable comparisons of our figures with those reported by the routine surveillance system is not possible. However, we have been able to compare our data with national data reported to the ECDC and with historical data.

Pertussis PCR results may be affected by antibiotic treatment lasting at least 5 days [33]. Although more than one third of pertussis cases had received an antibiotic before accessing the hospital – and were still PCR-positive – we may have missed additional cases receiving antibiotics and consequently underestimated the real incidence.

## Conclusion

Our study shows that in the participating hospitals’ catchment area, which roughly corresponds to the whole Lazio region, the incidence of pertussis in the first year of life has been high in recent years. Based on our assumptions, figures in the Lazio region in 2017 were almost twice as high as the mean European incidence of pertussis in young infants. It is likely that epidemic cycles will continue to occur in the absence of an increased coverage for the pertussis vaccine among pregnant women. In Italy, maternal immunisation remains a mainstay in pertussis prevention, particularly in preventing severe cases, which often occur in very young infants, and associated costs. To improve routine surveillance of pertussis, better pertussis diagnostic tools, including point-of-care tests in primary care, should be available to avoid misclassification of cases and to timely prescribe appropriate antibiotic therapy to prevent secondary cases.
